# Pencil-like Hollow Carbon Nanotubes Embedded CoP-V_4_P_3_ Heterostructures as a Bifunctional Catalyst for Electrocatalytic Overall Water Splitting

**DOI:** 10.3390/nano13101667

**Published:** 2023-05-18

**Authors:** Haiyang Chang, Zhijian Liang, Kun Lang, Jiahui Fan, Lei Ji, Kejian Yang, Shaolin Lu, Zetong Ma, Lei Wang, Cheng Wang

**Affiliations:** 1 Key Laboratory of Functional Inorganic Material Chemistry, Ministry of Education of the People’s Republic of China, Heilongjiang University, Harbin 150080, China; 1202864@s.hlju.edu.cn (H.C.); 1202775@s.hlju.edu.cn (Z.L.); 1202866@s.hlju.edu.cn (K.L.); 2201434@s.hlju.edu.cn (J.F.); 2 Jieyang Branch of Chemistry and Chemical Engineering Guangdong Laboratory, Jieyang 515200, China; jilei@gdut.edu.cn (L.J.); yangkj6@mail2.sysu.edu.cn (K.Y.); lushlin@gdut.edu.cn (S.L.); mazt@gdut.edu.cn (Z.M.)

**Keywords:** transition metal catalyst, oxygen evolution reaction, oxygen reduction reaction, zinc–air battery, electrocatalytic mechanism

## Abstract

Electrocatalytic water splitting is one of the most efficient ways of producing green hydrogen energy. The design of stable, active, and efficient electrocatalysts plays a crucial role in water splitting for achieving efficient energy conversion from electrical to hydrogen energy, aimed at solving the lingering energy crisis. In this work, CNT composites modified with CoP-V_4_P_3_ composites (CoVO-10-CNT-450P) were formed by carbonising a pencil-like precursor (Co_3_V_2_O_8_-H_2_O) and growing carbon nanotubes in situ, followed by in situ phosphorylation on the carbon nanotubes. In the HER electrocatalytic process, an overpotential of only 124 mV was exhibited at a current density of 10 mA cm^−2^. In addition, as an OER catalyst, a low overpotential of 280 mV was attained at a current density of 10 mA cm^−2^. Moreover, there was no noticeable change in the performance of the catalyst over a 90 h test in a continuous total water splitting experiment. The unique electronic structure and hollow carbon nanotube structure of CoVO-10-CNT-450P effectively increased the catalytic active sites, while also significantly improving the electrocatalytic activity. This work provides theoretical guidance for the design and synthetic route of high-performance non-precious metal electrocatalysts, and actively promotes the commercial application of electrochemical water splitting.

## 1. Introduction

With the continuous depletion of traditional fossil energy sources, the search for new, clean, efficient, and sustainable sources of energy remains one of the major challenges to the development of human civilisation [1,2,3]. Hydrogen is regarded as one of the most promising alternatives to fossil energy due to its advantages of high energy density, green environmental protection, renewability, and so on [4,5,6]. Currently, electrochemical water splitting is considered to be one of the mainstream technologies for hydrogen production due to its high stability, low pollution, and high efficiency, but the growing need for highly active and stable electrocatalysts to reduce the overpotential of hydrogen and oxygen precipitation reactions (OER) during water electrolysis has limited further developments in this field [7,8,9]. Enormous efforts have been devoted by researchers in recent years towards the synthesis of bifunctional electrocatalysts for the electrolysis of water. For the time being, noble-metal-based catalysts are the most widely used electrocatalysts for water splitting [10,11,12,13]. However, the scarcity of their constituent materials is a limitation to their application. Against this background, the development of cheap and efficient transition-metal-based materials are the most promising substitutes for noble-metal-based catalysts for industrial electrodes.

Non-noble transition metal compounds such as Fe, CO, and Ni have shown great promise in the efficient catalysis of HER and OER [14]. In general, the introduction of non-metallic atoms such as P, C and N breaks the periodicity of the original crystals, resulting in local changes to the electronic structure, which effectively changes the adsorption and resolution abilities of the catalysts, thus improving electrochemical performances [14,15,16,17]. Among them, cobalt phosphide (CoP) has become a promising catalyst for water splitting due to its low cost, excellent intrinsic activity and good stability during catalytic processes. However, the existing catalytic activity of CoP-based catalysts still falls short of the requirements for industrial production; hence, improvements are necessary. Bimetallic phosphide heterostructures have received significant attention in water splitting compared to single metal phosphide electrocatalysts. This is attributed to the existence of bimetallic phosphides that could adjust the electronic structure of heterostructures and the energetics of OER and HER intermediates. Vanadium and cobalt-based catalysts show low overpotential for water splitting electrocatalysts. Relatively speaking, Co-based phosphides have demonstrated excellent HER performance, while V-based phosphides display OER activity well. Therefore, it is a challenge to develop bimetallic vanadium and cobalt-based phosphides as bifunctional electrocatalysts with stable and uniform structures [9,11,15].

It is common knowledge that the catalytic activity of a catalyst is closely related to its morphology and composition. Therefore, the proper design of the composition and structure of a catalyst contributes to the adjustment of its electronic structure, which enhances charge and mass transfer and increases the active surface area. For example, Xu et al. [18] doped carbon in nanoporous CoP using an electrochemical dealloying method, and reported that its overpotential was only 30 mV when the current density was 10 mA cm^−2^. Hou et al. [19] restricted the growth of metal nanoparticles during a phosphating process with the strong coordination of two-dimensional Co-MOF nanosheets containing N, and the obtained small size cobalt-based phosphide showed high catalytic activities against HER and OER [14]. In addition to regulating the electronic structure of catalysts, the addition of active sites is also critical for the improvement of electrocatalytic performances. Recently, the improvements in the electrocatalytic activity of catalysts via the construction of superior morphologies, including nanowires, nanoflakes, nanospheres and nanotubes, have attracted great attention [20,21,22,23]. Among the several morphologies, hollow nanotubes have become a research hotspot in the field of electrocatalytic water splitting due to their large specific surface areas, rich adsorption/desorption sites and abundant electron transfer channels.

Herein, pencil-like hollow carbon nanotubes embedded CoP-V_4_P_3_ heterostructures as a bifunctional catalyst for electrocatalytic overall water splitting have been synthesized. Firstly, the orientated growth of carbon nanotubes (CNTs) was achieved by the carbonization of a pencil-like precursor (Co_3_V_2_O_8_·H_2_O), followed by in situ phosphating on the carbon nanotubes to form a CNT (CoVO-10-CNT-450P) modified by CoP-V_4_P_3_ composite. The results showed that the catalyst, CoVO-10-CNT-450P, which possessed a special three-dimensional morphology, showed high catalytic activity and excellent stability for HER and OER in 1 M KOH solution. When the current density of 10 mA cm^−2^ was attained, the required over-potentials for HER and OER were 124 mV and 280 mV, respectively. The unique electronic orbital structure that bimetal phosphide heterostructures serve can be as a highly active site to enhance electrocatalytic performance. The plentiful lone pair electrons and hollow d orbitals could manipulate the electronic states density, shift the d band centre, and adjust the electron spin state of heterostructures, thus promoting electrocatalytic activity [17]. At the same time, the overall water splitting device assembled with the catalyst only needed 1.66 V to reach a current density of 10 mA cm^−2^. In addition, the degradation rate (j ≈ 10 mA cm^−2^) of the catalyst remained almost unchanged after 90 h of continuous testing in overall water splitting. Hence, such an electrocatalyst, that is synthesized without the presence of noble metals, holds great promise in the design of efficient water splitting catalysts, thereby addressing the demand for effective stable catalysts for electrochemical overall water splitting.

## 2. Experimental Section

### 2.1. Materials

Ammonium metavanadate (NH_4_VO_3_ ≥ 99%), sodium hydroxide (NaOH ≥ 99.5%), cobalt chloride hexahydrate (CoCl_2_·6H_2_O ≥ 99%), dicyandiamide (C_2_H_4_N_4_ ≥ 99%), and sodium hypophosphite (NaH_2_PO_2_ ≥ 99.5%) were purchased from Sigma-Aldrich Chemicals Reagent Co, Ltd., St. Louis, MO, USA. All the chemicals were analytical grade and were used directly without further treatment. Deionized water was utilised for preparing the solutions and standards in this experiment.

### 2.2. Synthesis

Preparation of the precursor. Typically, 4.5 mmol of ammonium metavanadate was dissolved in 90 mL of deionized water, and heated to 80 °C. Then, 4.5 mmol of sodium hydroxide and 1.35 mmol of cobalt chloride hexahydrate were added to the solution. After fully stirring, the resulting solution was transferred to a 120 mL high-pressure reactor and allowed to react at 180 °C for 10 h, and the black powder at the bottom of the reactor was subsequently collected by centrifugation. After drying, a pencil-like cobalt vanadate precursor was collected and named CoVO-10. Two catalysts were further obtained by hydrothermal reaction for 5 and 12 h, and were named CoVO-5 and CoVO-12, respectively.

Preparation of CoVO-10-CNT. Briefly, 50 mg of the prepared precursor was measured out in a porcelain boat. Dicyandiamide with 5 times the quality of the precursor was taken into another porcelain boat as carbon source. The carbon source was placed upstream of the tubular furnace while the sample was placed downstream. The tubular furnace was heated to 900 °C at a rate of 5 °C min^−1^ in Ar atmosphere, and held for 2 h to afford CoVO-10-CNT. After cooling to room temperature, the downstream magnetic boat was removed and the samples were collected.

**Scheme 1:**
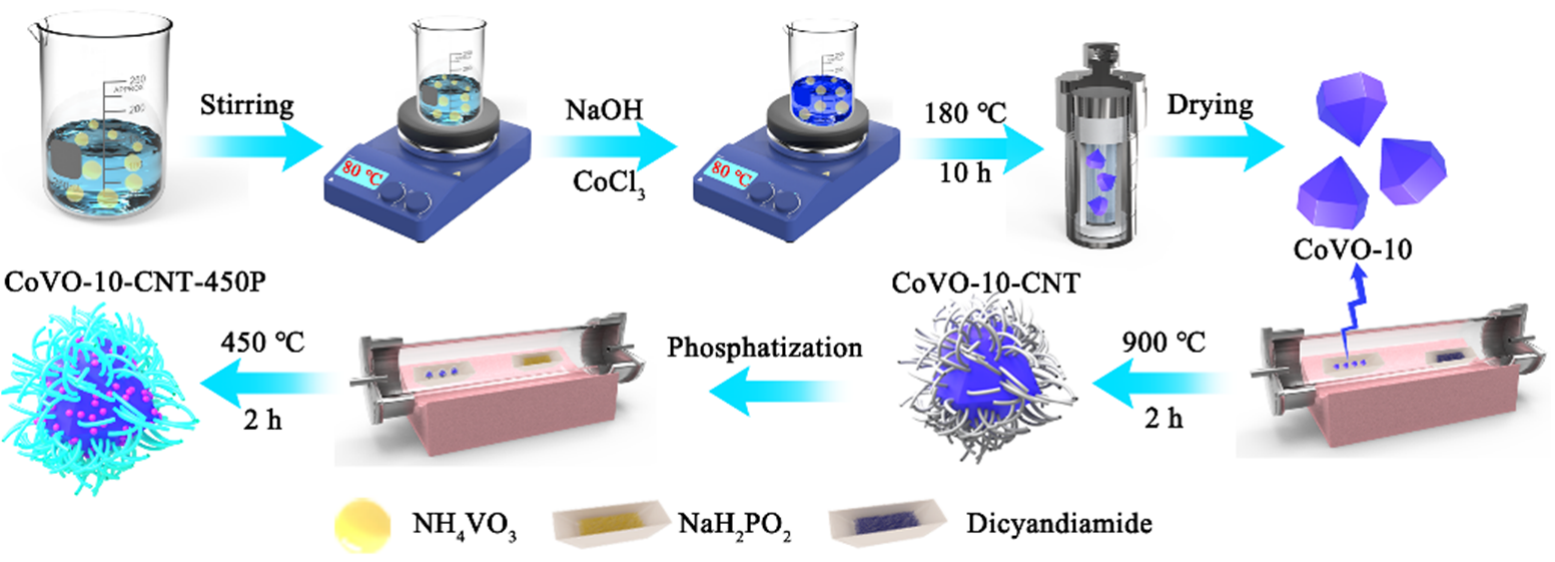
Synthetic schematic illustration of V-CoP-CNT.

Preparation of CoVO-10-CNT-450P. Briefly, 50 mg of the prepared CoVO-10-CNT was placed in a porcelain boat, and sodium hypophosphatite with 10 times its mass was measured out and placed in another porcelain boat as phosphorus source. The phosphorus source was placed upstream of the tube furnace while the sample was placed downstream. The tubular furnace was heated to 450 °C at the rate of 5 °C min^−1^ in Ar atmosphere, and held for 2 h to afford CoVO-10-CNT-450P. After cooling to room temperature, the downstream magnetic boat was removed and samples were collected. Similarly, the catalysts produced at 400 °C and 500 °C were named CoVO-10-CNT-400P and CoVO-10-CNT-500P, respectively. The catalyst synthesis procedure is illustrated in Scheme 1.

### 2.3. Characterizations

X-ray powder diffraction (XRD) measurements were taken on a D/max 2500 VL/PC diffractometer (Japan) equipped with graphite monochromatized Cu k_α_ radiation (*λ* = 1.54060 Å). Scanning electron microscopy (SEM) images were measured with JSM-7600F apparatus 6 at an acceleration voltage of 10 kV to examine the surface morphology of the catalysts. Transmission electron microscopy (TEM) images were recorded on JEOL-2100F instrument with an acceleration voltage of 200 kV. High-resolution TEM (HRTEM) images were obtained on an FEI Tecnai G2 F30 instrument with an acceleration voltage of 300 kV with elemental mapping. Energy dispersive X-ray spectroscopy (EDX) and most of the elemental mappings were performed on JSM-5160LV. X-ray photoelectron spectroscopy (XPS) was investigated on a scanning X-ray microprobe (PHI 5000 Verasa, Modify to Physical Electronics Industries Ulbridge ULAC-PHI, Inc., Shanghai, China) using monochromatized Al k_α_ radiation with a C1s peak at 284.8 eV used as internal standard. Nitrogen adsorption–desorption isotherms were performed at 77 K on a Quantachrome Instruments Autosorb AS-6B. ICP-OES (optical emission spectrometry) spectra were measured on an Agilent 725. XAFS was obtained on BL14W1 (Shanghai Synchrotron Radiation Facility, Shanghai, China) under transmission mode with a Si (111) double-crystal monochromator.

### 2.4. Electrochemical Testing

All electrochemical experiments were carried out on an electrochemical workstation (CHI 760E, Shanghai Chenhua Instrument Co., Ltd., Shanghai, China) using the conventional three-electrode system, where the prepared sample was used as the working electrode, SCE as the reference electrode, and a graphite rod as the counter electrode. The HER and OER activities of the catalysts were evaluated in 1 M KOH solution (pH = 13.7) by linear scanning voltammetry (LSV) at a scanning rate of 2 mV·s^−1^. According to E (vs. RHE) = E (vs. SCE) + 0.241 + 0.059 × pH, all electrode potentials measured relative to the reference potential were converted to potentials relative to the reversible hydrogen electrode (RHE). The stability of the studied electrocatalyst was evaluated by the i–t curve under constant current density. Electrochemical impedance spectroscopy (EIS) experiments were carried out at 10 mV amplitude in a frequency range from 1 × 10^5^ Hz to 0.1 Hz. The specific surface areas of all the prepared samples were compared by cyclic voltammetric scanning tests at different scanning rates (20 to 100 mV s^−1^) to evaluate the electric double layer capacitance (*C*_dl_). The full solution water electrolyser was prepared from a self-made single-chamber electrolyser. Foam nickel (1.5 cm × 1.5 cm) prepared with CoVO-10-CNT-450P acted as anode and cathode. Under similar experimental conditions and mass load (0.3 mg cm^−2^), both Pt/C/NF (cathode) and RuO_2_/NF (anode) were assembled in a water electrolysis tank for comparison.

## 3. Results and Discussion

### 3.1. Characterization of the CoVO-10-CNT-450P

**Figure 1:**
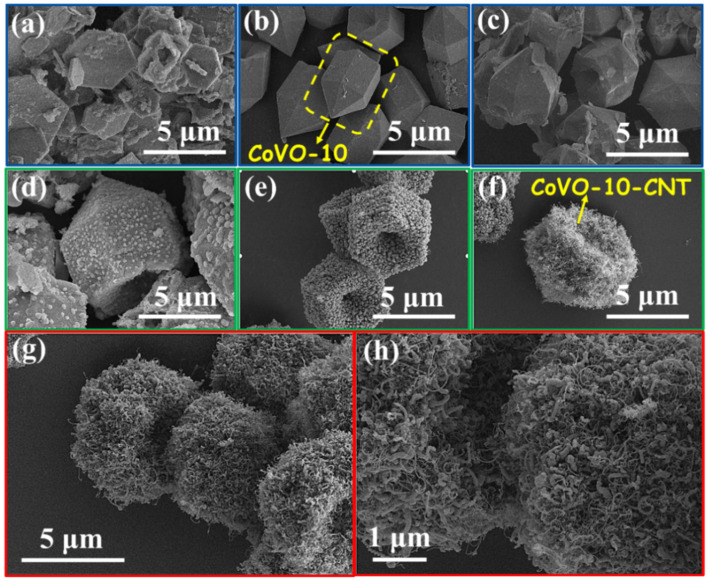
(**a**) SEM images of the calcined CoVO-5, (**b**) CoVO-10, (**c**) CoVO-12, and (**d**–**f**) CoVO-10-CNT at 600, 700, and 900 °C. (**g**,**h**) SEM images of CoVO-10-CNT-450P at different magnifications.

In order to explore the growth process of the “pencil-like” cobalt vanadate, we studied the morphology of the catalyst under different hydrothermal durations using SEM. The SEM analysis (Figure 1 and Figure S1) revealed that the hydrothermal time significantly affected the size and morphology of CoVO. After a hydrothermal reaction lasting 5 h, CoVO exhibited hexagonal nanosheet structures with irregular sizes and several intersections with each other. After 6 h, the pencil-like CoVO appeared with an initial size of 2.5 μm, but it was still accompanied by large amounts of nanoflakes. When the hydrothermal time was extended to 10 h, the nanosheet disappeared and the size of the pencil-like structure of CoVO gradually increased to 5 μm while being uniform. However, there were damages to the structural edges of CoVO, with the appearance of small fragments when the hydrothermal reaction was extended to 12 h. It can be inferred that cobalt vanadate first underwent an internal condensation reaction of its hexagonal nanoflakes to form a pyramid which grew from top to bottom, before finally forming a pencil-like structure. The formation of intermediates and precursors during the hydrothermal process may be responsible for directional crystal growth. The interaction between these intermediates and precursors can cause this growth pattern to occur. However, as the hydrothermal time is extended, the concentration and diffusion rate of intermediates and precursors will change, altering the rate and mechanism of crystal growth, and ultimately leading to the destruction of pencil-shaped structures.

In order to improve the conductivity of the material, CoVO-10 was used as the precursor for carbonisation treatment. Figure 1d–f and Figure S2 show that with increasing carbonization temperature, Co nanoparticles were precipitated on the surface of the pencil-like CoVO, which subsequently spread over the surface of the catalyst. When the temperature reached 750 °C, the in situ growth of irregular carbon nanotubes was observed on the metal nanoparticles. The surface of the catalyst was continuously coated with carbon nanotubes with increasing temperature, but the pencil-like structures of CoVO were still maintained. The sample was phosphated on the basis of carbonisation in order to further improve its performance. After phosphating (Figure 1g,h), the CoVO-10-CNT-450P still maintained the original CoVO-10-CNT morphology except for the thinning of the carbon nanotubes. The carbon nanotubes modified by metal nanoparticles provided rich active sites and increased the contact area between the catalyst and electrolyte, thus improving the catalytic activity and reaction kinetics. XRD tests were carried out on the samples at different carbonization temperatures (Figure S3), and the results confirmed the appearance of Co nanoparticles. The crystal phase of the precursor disappeared with increments in the carbonization temperature, and new diffraction peaks were observed at 44.2°, 51.5°, and 75.8°, corresponding to the (111), (200), and (220) crystal planes of Co (PDF # 1508-06), respectively. These possibly accelerated the carrier migration on the surface and provided more P-sites for the subsequent process. Moreover, it was observed that the crystallinity of the sample gradually increased with the increase in carbonisation temperature. The crystallinity decreased when the temperature reached 1000 °C, indicating a destruction of the structure.

**Figure 2:**
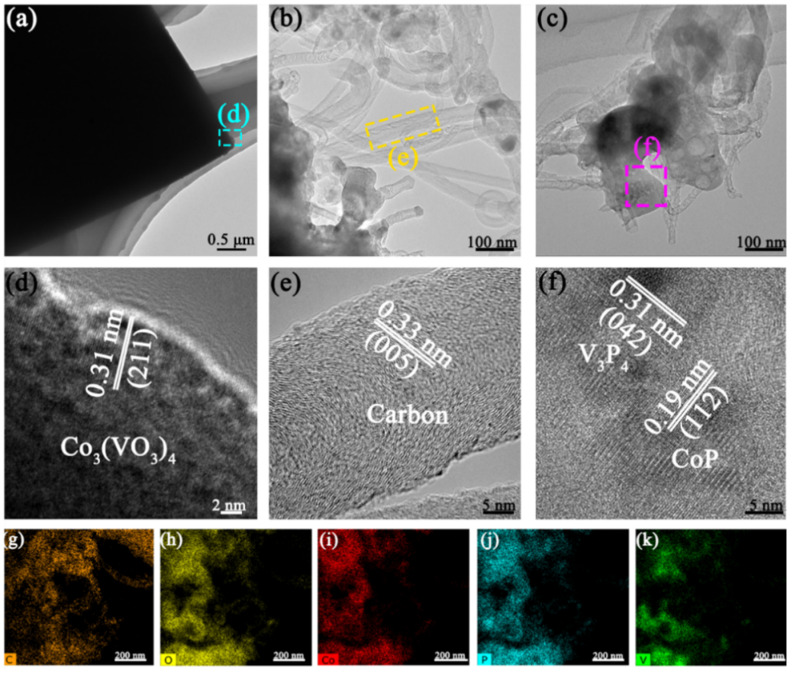
(**a**,**d**) CoVO-10, (**b**,**e**) CoVO-10-CNT, (**c**,**f**) CoVO-10-CNT-450P TEM at different magnifications, and (**g**–**k**) EDX images of C, Co, O, P and V elements.

The morphologies of the different catalysts were investigated by transmission electron microscopy (TEM). As shown in Figure 2a, the CoVO-10 showed pencil-like structures, which were consistent with the SEM test results. In addition, the crystal plane exposed on the surface appears to be the (211) crystal plane of CoVO-10, with lattice spacing of 0.31 nm (Figure 2d). Figure 2b shows the TEM image of CoVO-10-CNT. The results reveal the existence of a large number of irregular hollow tubular structures on the surface with lattice spacing of 0.33 nm, corresponding to the (005) crystal plane of carbon nanotubes (Figure 2e). This special three-dimensional structure is capable of increasing the specific surface area of the material to promote the diffusion of electrolyte and gas transmission. Figure 2c shows the TEM image after phosphating on the basis of CoVO-10-CNT. The high-resolution TEM image shows two distinctly different lattice stripes of 0.19 nm and 0.31 nm, attributed to CoP (112) and V_3_P_4_ (042), respectively (Figure 2f). The presence of CoP and V_3_P_4_ endows the catalyst with good OER and HER activities. The effective contact between the two phases can also facilitate a “synergistic effect” to improve the electrocatalytic performances.

The elemental mapping image from EDX shows the uniform distribution of the elements C, Co, O, P, and V in the CoVO-10-CNT-450P catalyst. The elemental composition and distribution on the surface of the sample were further analysed with EDX (Figure 2g,k). It was found that C, Co, O, P, and V elements were uniformly dispersed on the surface of the sample, which further proves that the sample was successfully phosphatised. To sum up, we have successfully prepared carbon nanotubes modified with CoP and V_3_P_4_ heterostructures on pencil-like cobalt vanadate.

**Figure 3:**
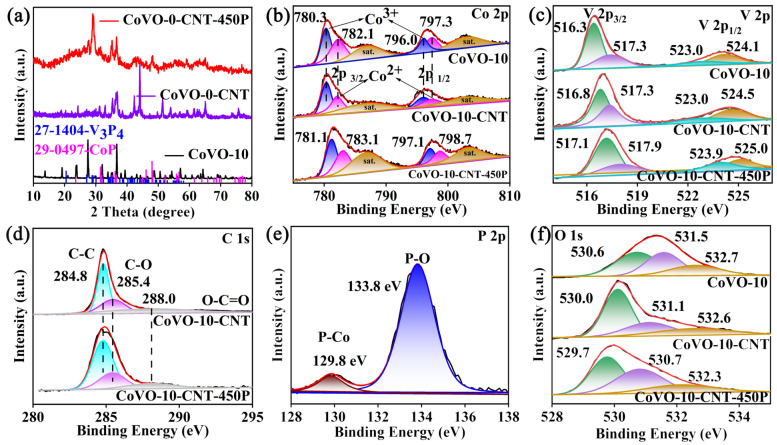
(**a**) XRD patterns of different electrodes. (**b**) XPS spectra of Co 2p, (**c**) C 1s, (**d**) V 2p, (**e**) P 2p, and (**f**) O 1s.

The crystalline phase composition of the samples was investigated using X-ray diffraction (XRD). The XRD spectra of the CoVO-10 precursors after carbonisation and phosphorylation treatments are shown in Figure 3a. For CoVO-10, the diffraction peaks at 19.1°, 23.6°, 24.0°, 27.4°, 32.1°, and 36.7° are attributed to the (101), (111), (300), (211), (400), and (221) crystal planes of Co_3_(VO_4_)_2_·H_2_O (PDF # 32-0291), respectively. For CoVO-10-CNT, in addition to the diffraction peak of the cobalt metal, the characteristic peaks at 26.3° and 43.8° correspond to the (002) and (102) crystal planes of graphite (PDF # 26-1077-carbon nanotubes) [24], indicating a high degree of crystallinity for the formed carbon nanotubes [25,26]. The characteristic peaks of the XRD spectra of CoVO-10-CNT-450P at 23.8°, 31.6°, 35.4°, 36.3°, 36.8°, and 48.4°are ascribed to the (101), (011), (200), (111), (102), and (202) crystal planes of CoP (PDF # 29-0497) [27]. Furthermore, the diffraction peaks at 29.2° and 29.4° are assigned to the (042) and (045) crystal planes of V_4_P_3_ (PDF#27-1404), indicating the formation of CoP-V_4_P_3_ composites. Raman spectroscopy was used to further characterise the structure of the sample. As shown in Figure S4, the two peaks at 332 cm^−1^ and 823 cm^−1^ are the asymmetric stretching vibration of the V-O-Co bond and the symmetric stretching vibration of the V-O bond, respectively [28]. In addition, the vibration peaks at 1374 cm^−1^ and 1598 cm^−1^ are attributed to the D band (sp^3^ carbon) and G band (sp^2^ carbon) of carbon, which, respectively, represent the degree of disorder and graphitisation of carbon on the material surface [28]. It is observed that I_D_/I_G_ increased after phosphorylation, indicating that the phosphorylation process produced more carbon defects and the increase in the number of defects facilitated the further enhancement of electrocatalytic activities [29,30].

X-ray photoelectron spectroscopy (XPS) was used to further investigate the elemental composition and chemical valency of the sample surface (Table S1). The high-resolution XPS spectrum of Co is shown in Figure 3b. For CoVO-10 and CoVO-10-CNT, the Co 2p spectrum can be divided into three peaks, corresponding to Co^3+^ (780.3 eV/796.0 eV), Co^2+^ (782.1 eV/797.3 eV), and the corresponding satellite peaks. Relatively speaking, the Co 2p spectrum of CoVO-10-CNT-450P shifted towards higher binding energy after phosphorylation, indicating that the valence state of Co was elevated during phosphorylation. Figure 3c shows the high-resolution XPS spectrum of V. For CoVO-10, the binding energy was 516.3 eV, the peak at 523.0 eV is attributed to V^4+^, and the peaks at 517.3 eV and 523.0 eV are attributed to V^5+^. Compared to the precursors, the V 2p spectrum shifted to the higher energy direction, indicating that V lost electrons in both processes [31]. Three peaks can be observed in the 1s spectrum of C (Figure 3d), of which the peak at 284.8 eV is the characteristic peak of the C-C bond, while the peaks at 285.4 eV and 288.0 eV are the characteristic peaks of C-O and O-C=O, respectively [32].

Figure 3e shows the 2p high-resolution XPS spectrum of P, and the peak at 133.7 eV belongs to the P–O bond, while that at 130.0 eV belongs to P–M [33,34], proving the existence of metal phosphide species. The introduction of the P atom into the metal lattice could slightly increase the length of the metal–metal bond. This slight modification weakens the interaction between metal atoms, leading to the reduction of the metal atom’s d band and further improving the electron density near the fermi level, thereby enhancing the electrocatalytic performance. Three characteristic peaks are shown in the O 1s high-resolution XPS spectrum (Figure 3f), and they, respectively, belong to the lattice oxygen (530.6 eV), vacancy oxygen (531.5 eV), and chemisorption oxygen (532.7 eV) [35,36]. Notably, for carbonisation as well as phosphorylation, the characteristic peaks of the oxygen element shifted towards the lower binding energy direction, indicating that the electron flow of the Co and V elements was towards O, further confirming that the internal electronic structure can be modulated by carbonization as well as phosphorylation. The nitrogen adsorption–desorption isotherm is shown in Figure S5. From the figure, it can be seen that all samples exhibited a typical type IV adsorption isotherm, indicating the presence of a large number of mesopores and micropores on the surface of the catalyst [37,38]. The corresponding Brunauer–Emmett–Teller (BET) specific surface area was calculated according to the isotherm. The results show that the BET specific surface area of CoVO-10 was 1.6 m^2^ g^−1^, which is much lower than those of CoVO-10-CNT (34.1 m^2^ g^−1^) and CoVO-10-CNT-450P (23.9 m^2^ g^−1^). These results indicate that the formation of hollow carbon nanotubes greatly increased the specific surface area of the material, thereby facilitating mass and charge transfer and increasing electrocatalytic activity.

**Figure 4:**
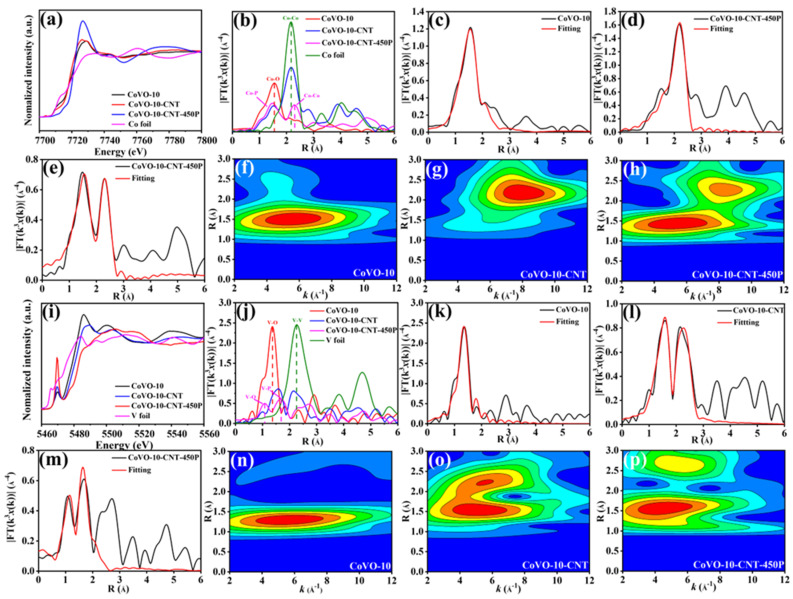
(**a**) Normalisation Co K edge XANES. (**b**)Fourier transform EXAFS spectra corresponding to CoVO-10, CoVO-10-CNT, CoVO-10-CNT-450P and Co-foil. (**c**–**e**) Co K-edge EXAFS fitting analysis corresponding to CoVO-10, CoVO-10-CNT, and CoVO-10-CNT-450P. (**f**–**h**) Co K-edge WT-EXAFS contour map of all comparison samples. (**i**) Normalised V K edge XANES. (**j**) Fourier transform EXAFS spectra corresponding to CoVO-10, CoVO-10-CNT, CoVO-10-CNT-450P, and V foil. (**k**–**m**) V K-edge EXAFS fitting analysis corresponding to CoVO-10, CoVO-10-CNT, and CoVO-10-CNT-450P. (**n**–**p**) V K-edge WT-EXAFS contour map of all comparison catalysts.

In addition, the coordination environment and bonding state of Co and V atoms in CoVO-10-CNT-450P, as well as at the Co K-edge and V K-edge, were studied with X-ray absorption near-edge structure (XANES) and extended X-ray absorption fine structure (EXAFS) measurements. From the Co XANES spectrum (Figure 4a), it was observed that the adsorption edge of CoVO-10-CNT-450P shifted towards higher energies relative to those of CoVO-10 and CoVO-10-CNT. This indicates that the valency of Co in CoVO-10-CNT-450P was higher than that of CoVO-10 and CoVO-10-CNT, which is consistent with the reported XPS results. Additionally, in the EXAFS spectrum shown in Figure 4b, CoVO-10-CNT-450P displayed two characteristic peaks at 1.5 and 2.3 Å, which belonged to the Co-P and Co-Co bonds, respectively [39,40,41,42]. Moreover, the V K edge XANES spectra of CoVO-10-CNT-450P showed that the edge position of CoVO-10-CNT-450P (Figure 4i) also shifted to the higher energy direction compared with that of the tested reference samples, and this indicates that the valence state of V in CoVO-10-CNT-450P was higher [43]. In Figure 4j, the V K-edge EXAFS of CoVO-10-CNT-450P was very different from that of V foil and CoVO-10. Two main peaks around 1.07 Å and 1.7 Å were clearly observed, and they correspond to the scattering paths of V-O and V-P of CoVO-10-CNT-450P. It is noteworthy that the peak intensity of CoVO-10-CNT-450P at the same position with the V foil at 2.26 Å was very weak, indicating that V was essentially completely phosphorylated. The wavelet-transformed X-ray absorption fine spectrum (WT-XAFS) contour of CoVO-10-CNT-450P displayed two main maxima around 5 and 8 Å^−1^, which were related to Co-P and Co-Co bonds, respectively (Figure 3f–h). As shown in Figure 3n–p, CoVO-10-CNT-450P and its control had a similar maximum strength of around 5 Å^−1^, which was attributed to the V-O coordination bond. In addition, CoVO-10-CNT-450P and CoVO-10-CNT manifested an extra strength around 6 Å^−1^, and this corresponds to the V-V bond, which is consistent with the EXAFS fitting data. Interestingly, the contour map of CoVO-10-CNT-450P revealed a slight shift compared with the maps of CoVO-10 and CoVO-10-CNT, which proves that charge transfer occurred between the Co and V atoms.

### 3.2. The HER Catalytic Activity of CoVO-10-CNT-450P

**Figure 5:**
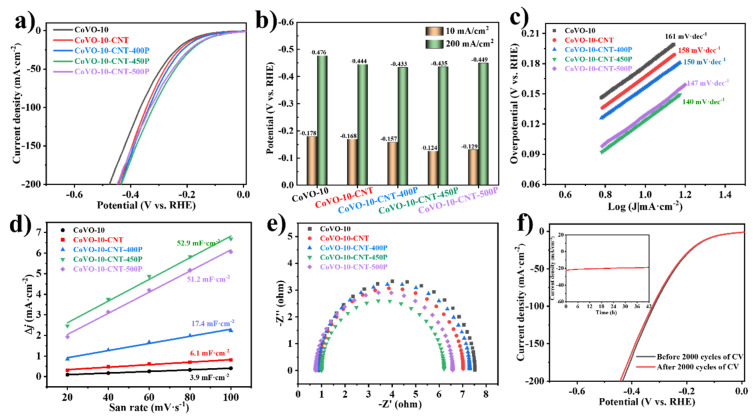
HER performance tested in 1 M KOH electrolyte: (**a**) LSV curves, (**b**) overpotentials at 10 and 200 mA cm^−2^, (**c**) Tafel curve, (**d**) ECSA values, and (**e**) Nyquist curves of different catalysts. (**f**) Stability test of CoVO-10-CNT-450P.

To evaluate the catalytic performance of CoVO-10-CNT-450P, its activity was tested in 1 M KOH using a standard three-electrode system (with the carbon rod and SCE electrode as counter electrode and reference electrode, respectively). All measured currents were corrected for the effect of ohmic resistance. The curves for the linear scanning voltammetry (LSV) tests are shown in Figure 5a. CoVO-10-CNT-450P showed comparatively better HER activity than the other samples, with overpotentials of only 124 and 435 mV required to achieve current densities of 10 and 200 mA cm^−2^, which was sufficiently superior to those of CoVO-10-CNT-400P (157 and 433 mV) and CoVO-10-CNT-500P (129 and 449 mV) (Figure 5b). This revealed the relationship between phosphorylation temperature and electrocatalytic activity. In addition, CoVO-10 and CoVO-10-CNT performed poorly in the HER investigation, and the overpotential values at 10 mA cm^−2^ were, respectively, 178 and 168 mV, indicating that the CoP-V_4_P_3_ composite played an important role in HER. The Tafel slope is an important tool for clarifying the reaction kinetics of materials. The Tafel slope value was calculated based on the Tafel equation (*η* = a + b log|*j*|), where b is the Tafel slope and j is the current density. As shown in Figure 5c, the Tafel slope of CoVO-10-CNT-450P was 140 mV·dec^−1^, which was lower than that of the compared samples, indicating that the catalyst modified by the CoP-V_4_P_3_ composite exhibited rapid HER reaction kinetics. These results show that the HER process of CoVO-10-CNT-450P follows the Volmer–Heyrovsky mechanism.

The electrochemical active surface area (ECSA) was determined to have a relationship with the activity of the electrocatalyst, while ECSA was proportional to the double layer capacitance (*C*_dl_). The catalyst was tested using cyclic voltammetry (CV) in the range of 0–0.2 V at different sweep rates, as shown in Figure S6. Comparatively, CoVO-10-CNT-450P exhibited the largest *C*_dl_, of 52.9 mF cm^−2^ (Figure 5d), signifying that its surface had much more active sites. This was due to the hollow carbon nanotube structure obtained after phosphorylation, which provides more active sites. The EIS spectra show that CoVO-10-CNT-450P had the least radius compared with other catalysts, and it had the least charge transfer resistance of *R*_ct_ = 2.36 Ω (Figure 5e, Table S2). These were attributed to the high electrical conductivity of the carbon nanotubes and the rapid charge transfer between the different components of the catalyst. In addition to the catalytic activity, long-term stability is also an important criterion for evaluating the performances of catalysts. As shown in Figure 5f and Figure S7, the polarisation curve of the CoVO-10-CNT-450P catalyst after 2000 consecutive CV cycles was observed to almost completely overlap with the curve recorded before the cycle (Figure S9). The decrease in current density within 42 h can be ignored, as there was only a 3.2% loss rate of current at 1.77 mV, which is further evidence of the excellent cyclic stability of the catalyst (Figure 5f).

**Figure 6:**
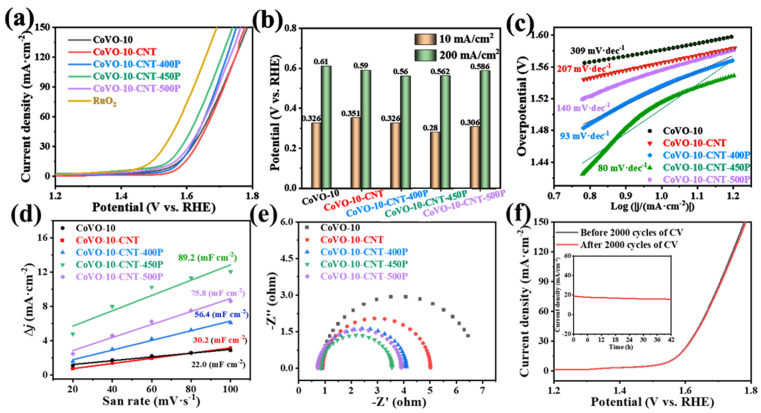
OER performance tested in 1 M KOH electrolyte: (**a**) LSV curves, (**b**) overpotentials of different catalysts at 10 and 200 mA cm^−2^, (**c**) Tafel curves, (**d**) ECSA values, and (**e**) Nyquist curves of different catalysts. (**f**) Stability test of CoVO-10-CNT-450P.

Similarly, the OER performance of CoVO-10-CNT-450P was investigated using the same three-electrode system in 1M KOH. Figure 6a shows the LSV curve of the iR-corrected catalysts. Similarly, the CoVO-10-CNT-450P sample also showed the best OER activity, requiring only 280 and 562 mV to reach current densities of 10 and 200 mA cm^−2^, respectively. In contrast, CoVO-10, CoVO-10-CNT, CoVO-10-CNT-400P, CoVO-10-CNT-500P, and RuO_2_, respectively, required 326, 351, 326, 306, and 170 mV to drive a current density of 10 mA cm^−2^. It is noteworthy that there was almost no difference between the LSV of CoVO-10 and CoVO-10-CNT. This is because the presence of carbon nanotubes on OER activity is negligible, meaning that the excellent OER performance of CoVO-10-CNT-450P originated from the formation of CoP-V_4_P_3_ composites after phosphating. Figure 6c shows the Tafel slopes of the catalysts. The Tafel slopes were calculated from the LSV curves to assess the dynamics of the catalyst (Figure 6c). The Tafel slope of CoVO-10-CNT-450P was 80 mV dec^−1^, which is less than those of other catalysts such as CoVO-10 (309 mV dec^−1^) and CoVO-10-CNT (207 mV dec^−1^), indicating that CoVO-10-CNT-450P displayed faster OER kinetics. From Figure 6d, the *C*_dl_ values of CoVO-10, CoVO-10-CNT, CoVO-10, CoVO-10-CNT-450P, and CoVO-10-CNT-500P were 30.2, 22.0, 56.4, 75.8, and 89.2 mF·cm^−2^, respectively. Relatively speaking, CoVO-10-CNT-450P observably had the largest *C*_dl_, indicating an obvious advantage over the other catalysts in terms of active surface area and active site.

The Nyquist plots shown in Figure 6e further reveal the good charge transfer resistance of CoVO-10-CNT-450P, owing to the lowest *R*_ct_ value (Table S3). In addition, the CoVO-10-CNT-450P composite catalyst also exhibited an excellent OER stability. As shown in Figure 6f, the LSV curve showed no changes after 2000 CV cycles, indicating the high stability of CoVO-10-CNT-450P with only a 1.6% current density loss rate at 1.77 mV. After the stability test, no peak shifts or significant crystallinity changes could be observed in the XRD pattern (Figure S10). CV curves of various catalysts at 20–100 mV/s are shown in Figures S8 and S11. Before and after the test, the structure of the CoVO-10-CNT-450P was not damaged in a large area, but still maintained the pencil-like morphology fully wrapped by nanotubes, which further confirmed the excellent stability of the CoVO-10-CNT-450P. The above results show that the CoVO-10-CNT-450P exhibited a bifunctional catalytic activity. Importantly, the synthetic CoVO-10-CNT-450P displayed great industrial potential compared with other recently reported bifunctional electrocatalysts (Table S4).

**Figure 7:**
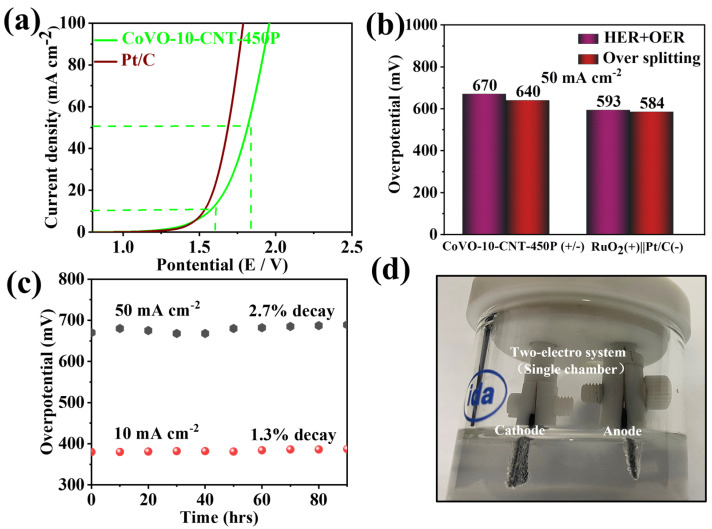
(**a**) I–V curve of overall water splitting using CoVO-10-CNT-450P electrode. (**b**) The overpotentials comparison of two-electrode and three-electrode systems. (**c**) The stability of CoVO-10-CNT-450P electrode at the constant current of 10 and 50 mA cm^−2^ for 90 h. (**d**) Overall water splitting photo of the two-electrode system assembled by CoVO-10-CNT-450P.

Considering its excellent electrocatalytic performance for HER and OER, the CoVO-10-CNT-450P catalyst was integrated into a double-electrode alkaline electrolyte (without membrane) as the cathode and anode, and a full water splitting performance was further studied (Figure 7d). As shown in Figure 7a,b, the CoVO-10-CNT-450P catalyst exhibited good performance in the overall water splitting investigation. It needed 1.66 and 1.87 V voltages to attain the current densities of 10 and 50 mA cm^−2^, respectively. The voltage was significantly lower than that of CoVO-10 and slightly higher than that of Pt/C/RuO_2_ assembled for the overall water splitting device (Figure 7a). More importantly, the electrolytic cell assembled based on CoVO-10-CNT-450P showed good stability at current densities of 10 and 50 mA cm^−2^, with a decay rate of only 1.3 and 2.7% after continuous stability testing for 90 h. The above results showed that the CoVO-10-CNT-450P composite catalyst had a bifunctional activity. Notably, the water splitting voltage of CoVO-10-CNT-450P was lower than most of the advanced catalysts, indicating the greater potential for industrial application (Table S5).

## 4. Conclusions

In summary, pencil-like cobalt vanadate was hydrothermally synthesized in this work and subsequently carbonised and phosphated to afford a CoP-V_4_P_3_ composite. Subsequently, a CoVO-10-CNT-450P catalyst with high catalytic activity for both HER and OER was obtained by adjusting the phosphorylation temperature. At a current density of 10 mA cm^−2^, the overpotentials for HER and OER were 124 mV and 280 mV, respectively, with good stability. Moreover, a current density of 10 mA cm^−2^ was achieved with only 1.66 V when two identical CoVO-10-CNT-450P catalysts were used as cathode and anode to assemble an electrochemical total hydrolysis unit. The excellent performance of the CoVO-10-CNT-450P catalyst may be attributed to the synergistic catalysis between different components, as well as the special pencil-like and hollow carbon nanotube structures which provided large electrocatalytic surface area and high electrical conductivity. This work offers a meaningful pathway for the integration of different components as heterostructures, and the construction of efficient and robust integrated water splitting catalysts.

## Data Availability

The dataset used in this study will be available upon reasonable request to the corresponding author.

## Supplementary Materials

The following supporting information can be downloaded at: https://www.mdpi.com/article/10.3390/nano13101667/s1. Figure S1. SEM images of CoVO synthesized by different hydrothermal reaction times. Figure S2. SEM images of CoVO-10-CNT synthesized by different vapor deposition calcination temperatures. Figure S3. XRD patterns of the composites derived from CoVO-10 carbonized at different temperatures. Figure S4. N2 adsorption-desorption isotherms and corresponding BET surface areas of (a) CoVO-10, (b) CoVO-10-CNT and (c) CoVO-10-CNT-450P. The illustration shows the pore size distribution of the corresponding sample. Figure S5. Raman spectra of different samples. Figure S6. CV curves of different samples tested at 20–100 mV·s-1 in the potential range of 0–0.2V. Figure S7. CV cycles of CoP/V3P4-CNT tested at the potential range of –1.6~–0.6 V. Figure S8. CV curve of different catalysts tested at 20–100 mV–1 in the potential range of 1.1–1.2 V. Figure S9. CV cycles of CoP/V3P4-CNT test in the potential range of 0–0.8 V. Figure S10. XRD pattern of CoVO-10-CNT-450P after stability test. Figure S11. (a,b) SEM images of CoVO-10-CNT-450P after stability test. Table S1. The element contents of Co, V, O, C and P elements in CoVO-10-CNT CoVO-10-CNT-450P determined by XPS analyses. Table S2. The resistance values for HER calculated from the EIS results of Figure 5e. Table S3. The resistance values for OER calculated from the EIS results of Figure 6e. Table S4. Performance comparison of recent non-noble metal bifunctional HER and OER electrocatalysts tested in 1.0 M KOH electrolyte. Table S5. Comparison the overall water splitting performance of CoVO-10-CNT-450P with previous reported superior bifunctional electrocatalysts tested in 1.0 M KOH. References [22,44,45,46,47,48,49,50,51,52,53,54,55,56,57,58,59,60,61,62,63] are cited in the supplementary materials.
