# Supplementary material for: Pencil-like Hollow Carbon Nanotubes Embedded CoP-V4P3 Heterostructures as a Bifunctional Catalyst for Electrocatalytic Overall Water Splitting
Source: Nanomaterials (Basel). 2023 May 18;13(10):1667. doi: 10.3390/nano13101667 (PMC10222086; doi:10.3390/nano13101667)
Supplement: Supplementary file 1 [file nanomaterials-13-01667-s001.zip › nanomaterials-2372817-supplementary.pdf]

Supporting Information

Pencil-like Hollow Carbon Nanotubes Embedded $CoP-V_4P_3$ Heterostructures as a Bifunctional Catalyst for Electrocatalytic Overall Water Splitting

Haiyang Chang [1,2,†], Zhijian Liang [1,†], Kun Lang [1], Jiahui Fan [1], Lei Ji [2], Kejian Yang [2], Shaolin Lu [2], Zetong Ma [2], Lei Wang [1,*] and Cheng Wang [1,2,*]

1 Key Laboratory of Functional Inorganic Material Chemistry, Ministry of Education of the People's Republic of China, Heilongjiang University, Harbin 150080, China; 1202864@s.hlju.edu.cn (H.C.); 1202775@s.hlju.edu.cn (Z.L.); 1202866@s.hlju.edu.cn (K.L.); 2201434@s.hlju.edu.cn (J.F.)

2 Jieyang Branch of Chemistry and Chemical Engineering Guangdong Laboratory, Jieyang 515200, China; jilei@gdut.edu.cn (L.J.); yangkj6@mail2.sysu.edu.cn (K.Y.); lushlin@gdut.edu.cn (S.L.); mazt@gdut.edu.cn (Z.M.)

* Correspondence: wanglei0525@hlju.edu.cn (L.W.); wangcheng@hlju.edu.cn (C.W.)

† These authors contributed equally to this work.

SEM images of CoVO

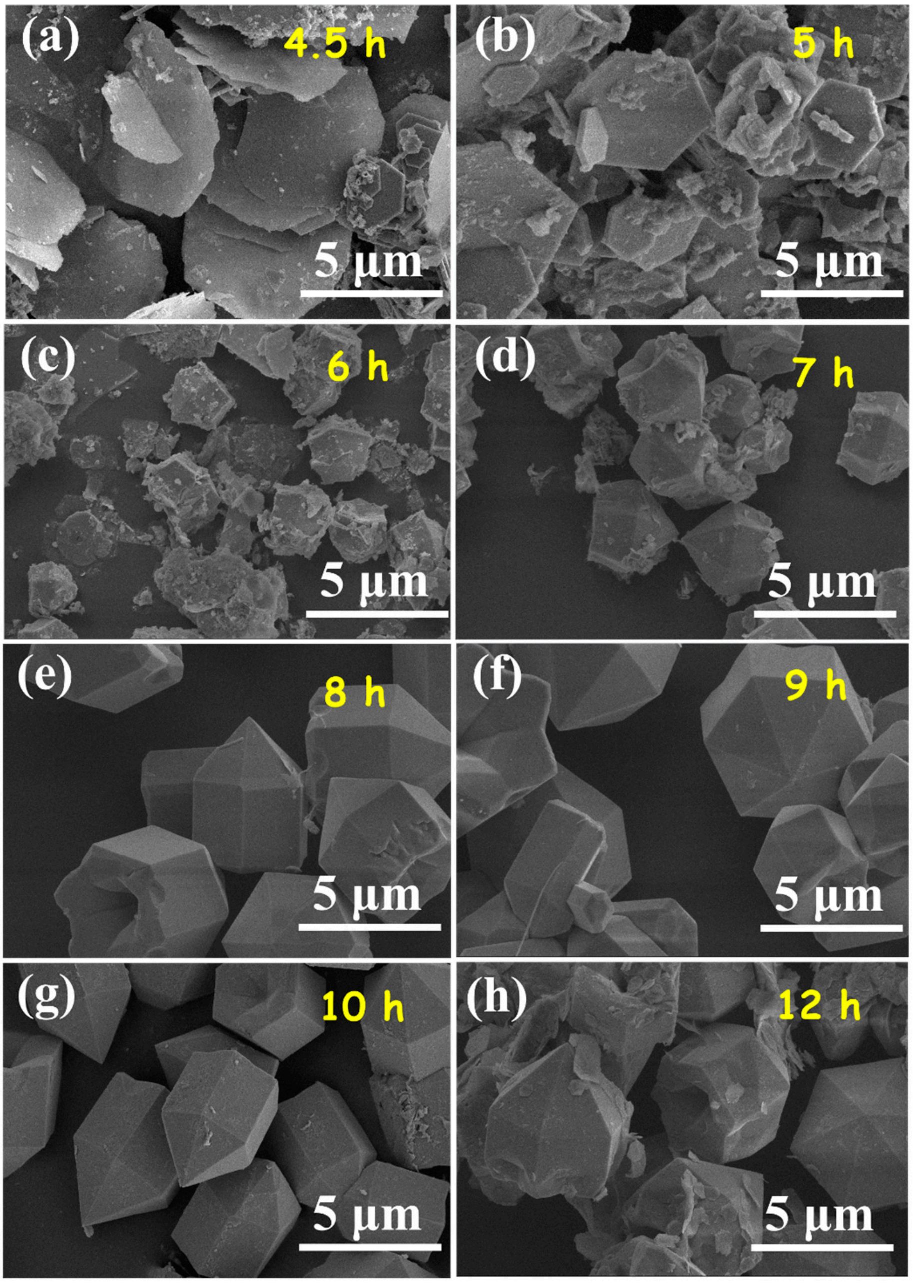

Figure S1. SEM images of CoVO synthesized by different hydrothermal reaction times.

SEM images of CoVO-10-CNT

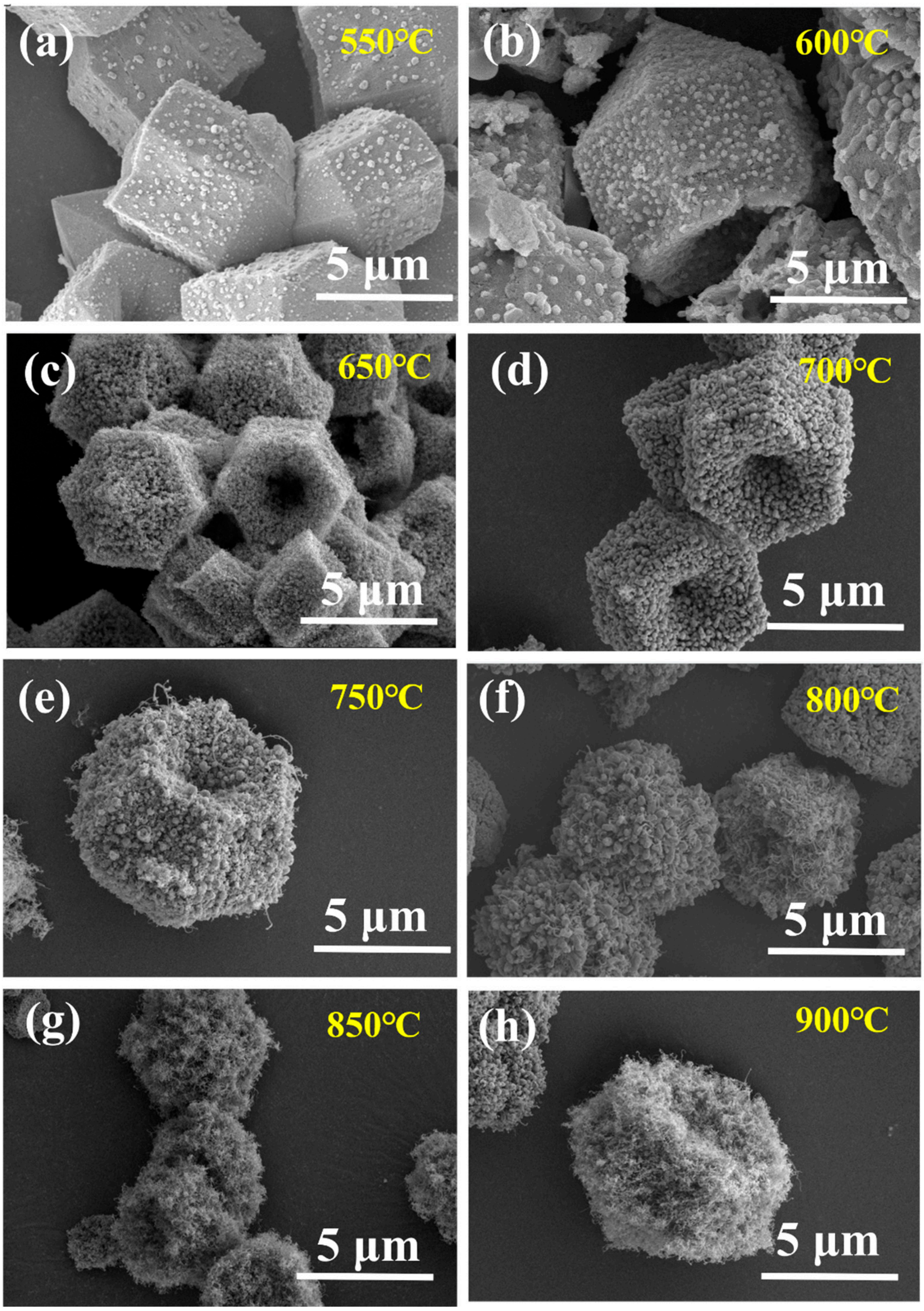

Figure S2. SEM images of CoVO-10-CNT synthesized by different vapor deposition calcination temperatures.

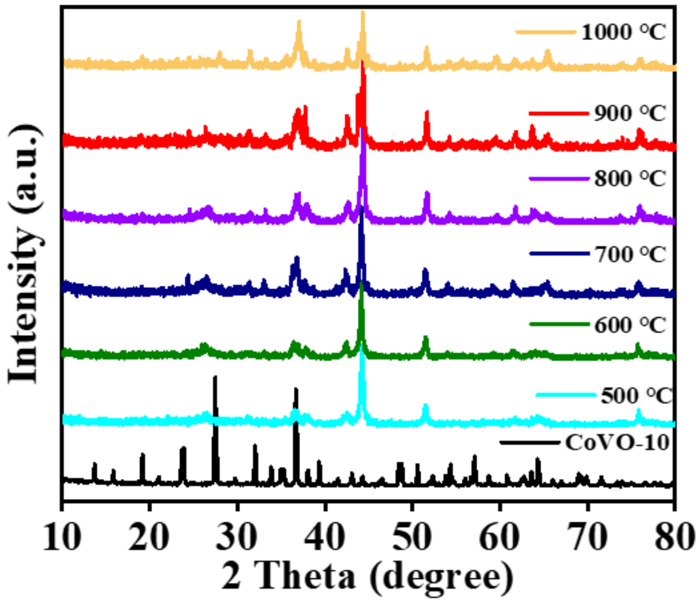

Figure S3. XRD patterns of the composites derived from CoVO-10 carbonized at different temperatures.

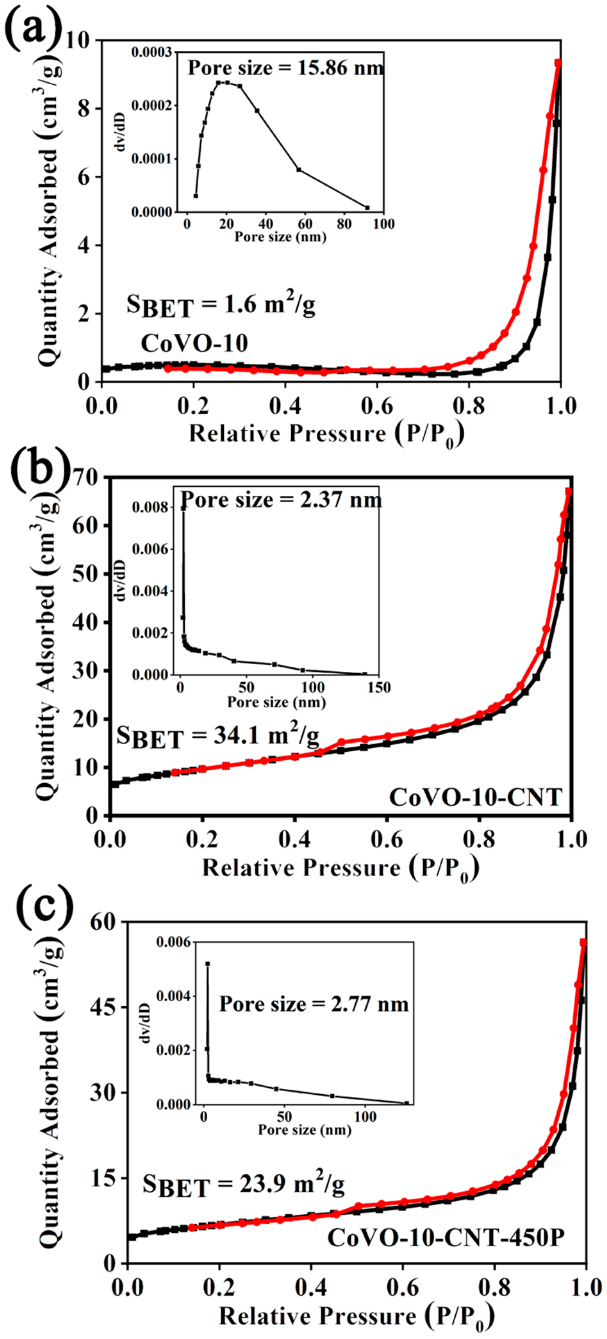

Figure S4. N_2 adsorption-desorption isotherms and corresponding BET surface areas of (a) CoVO-10, (b) CoVO-10-CNT and (c) CoVO-10-CNT-450P. The illustration shows the pore size distribution of the corresponding sample.

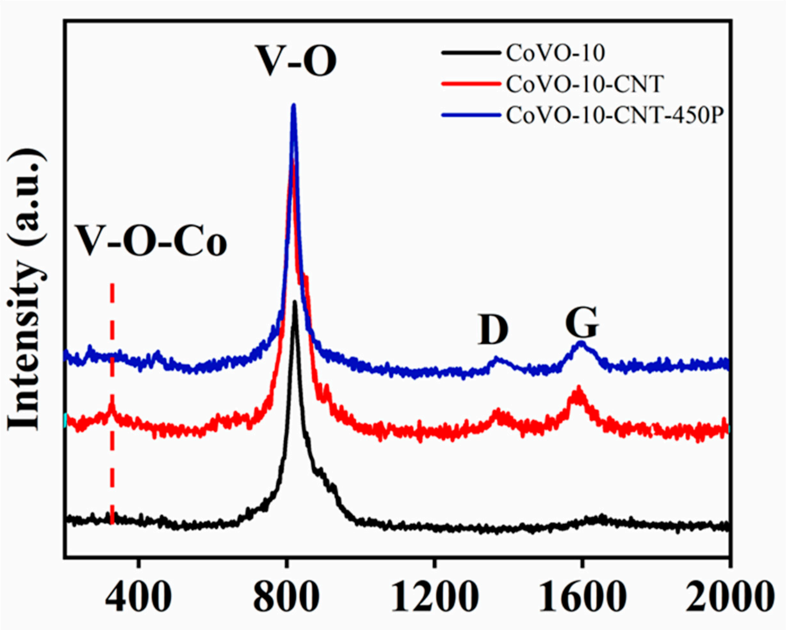

Figure S5. Raman spectra of different samples.

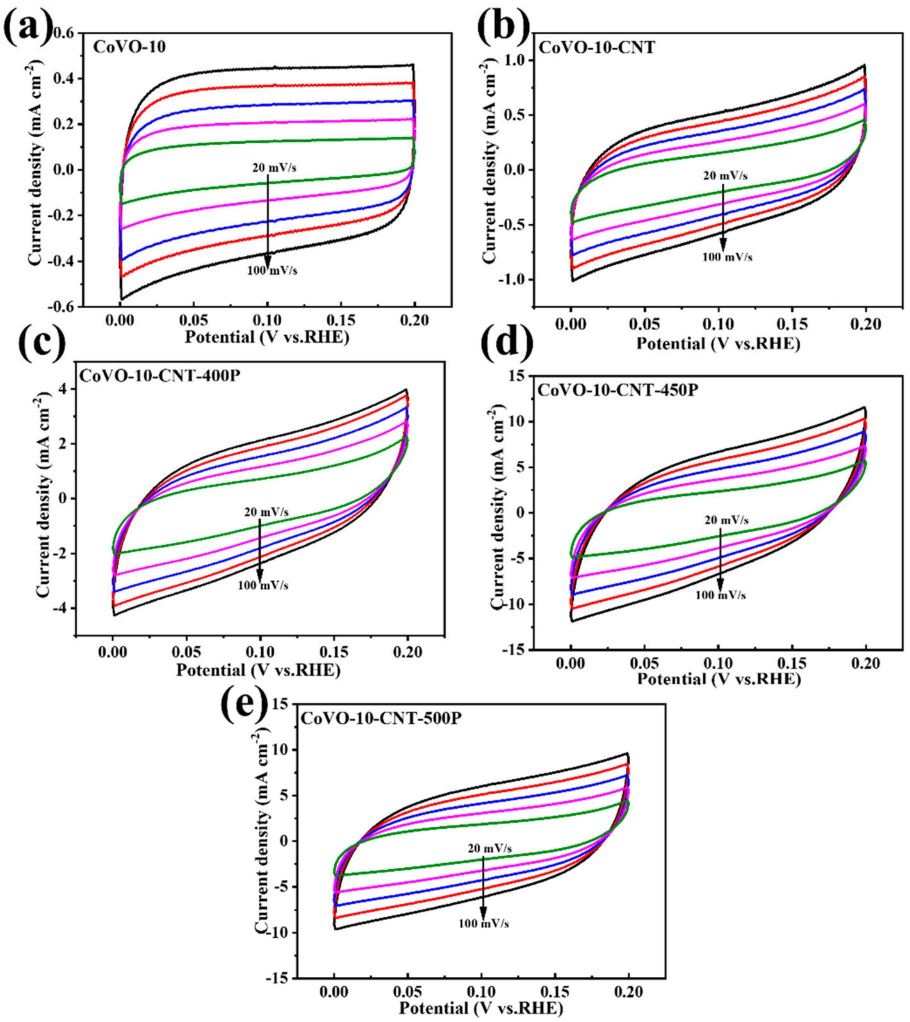

Figure S6. CV curves of different samples tested at 20–100 mV·s^{-1} in the potential range of 0–0.2V.

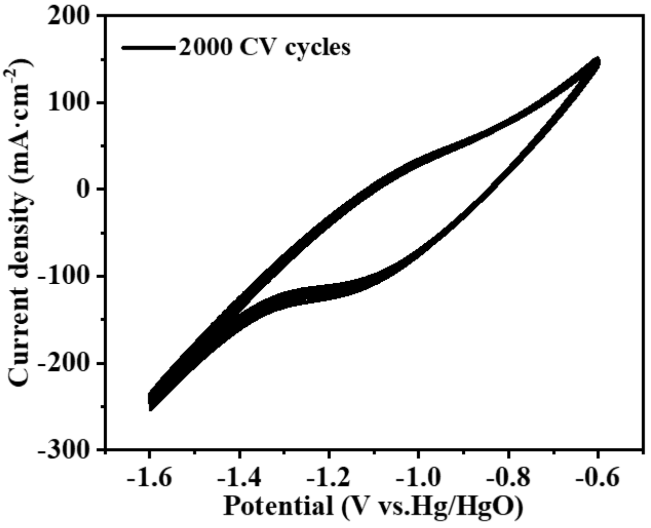

Figure S7. CV cycles of CoP/V_3P_4-CNT tested at the potential range of –1.6~–0.6 V.

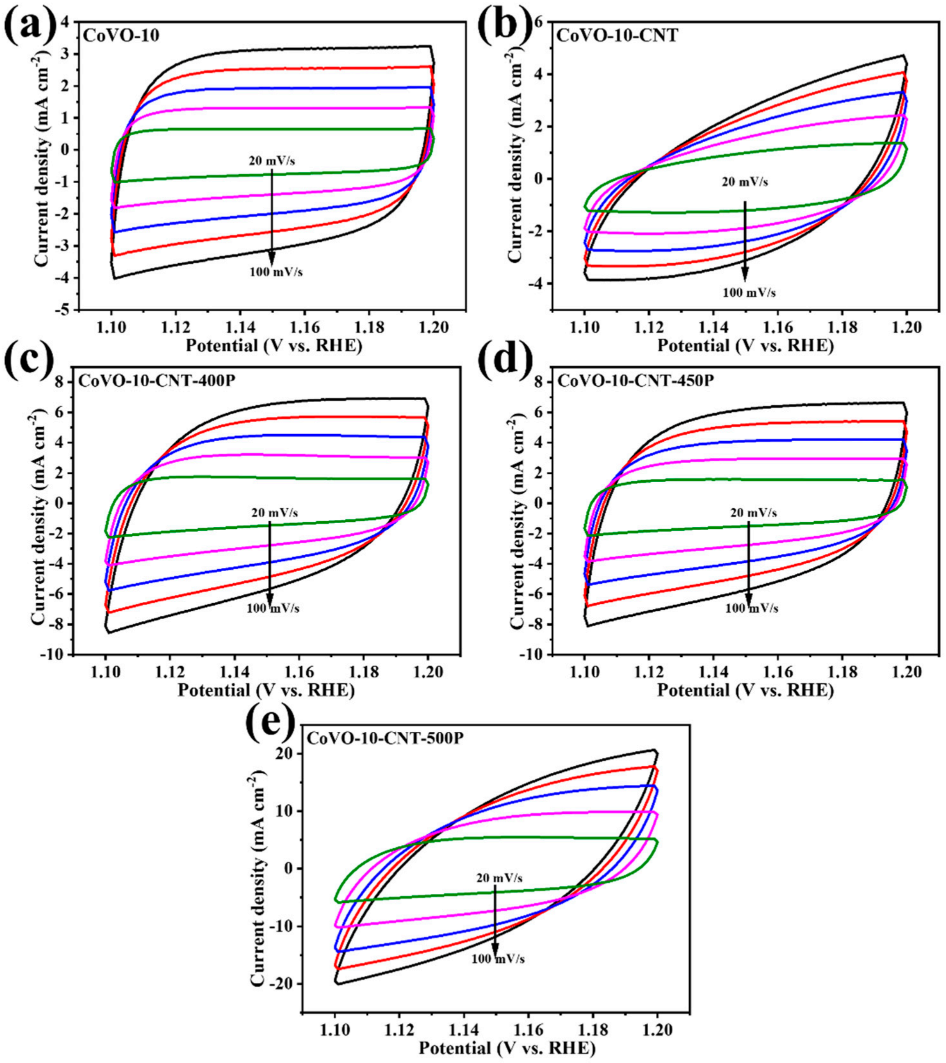

Figure S8. CV curve of different catalysts tested at 20–100 mV^{-1} in the potential range of 1.1–1.2 V.

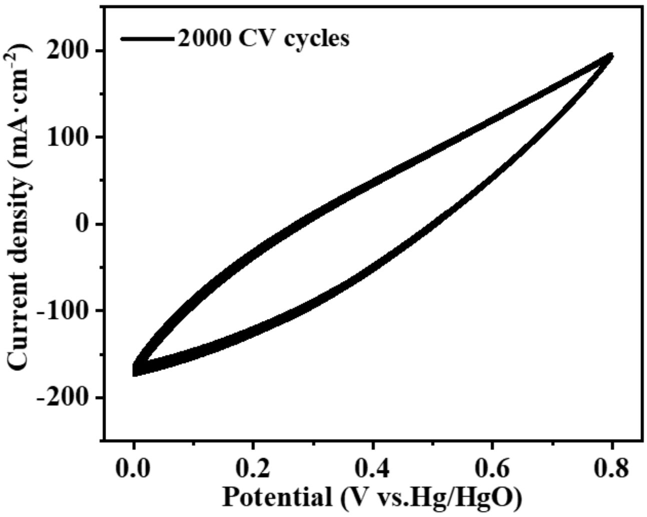

Figure S9. CV cycles of CoP/V_3P_4-CNT test in the potential range of 0–0.8 V.

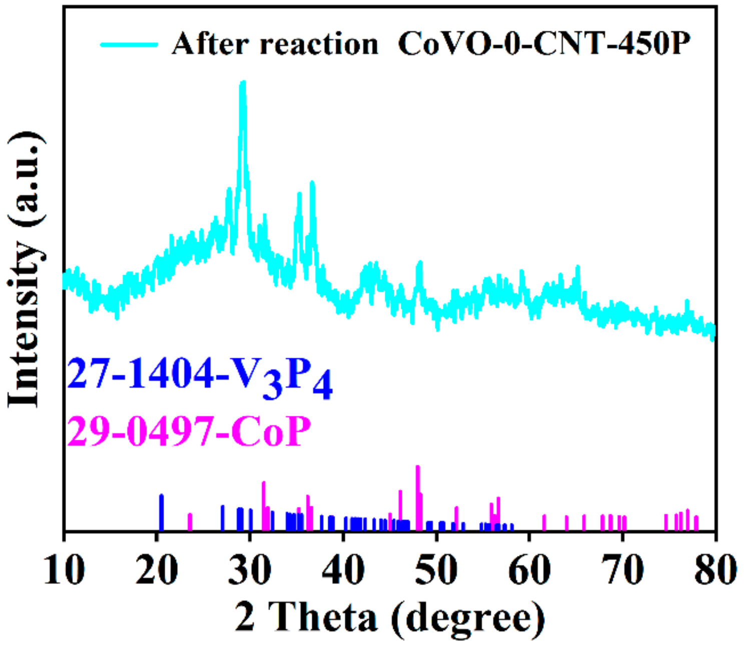

Figure S10. XRD pattern of CoVO-10-CNT-450P after stability test.

SEM images of CoVO-10-CNT-450P

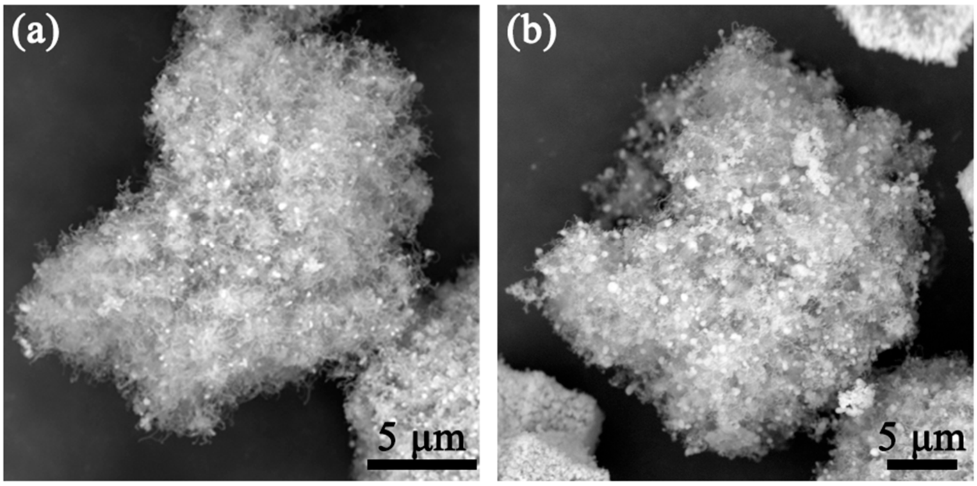

Figure S11. (**a**,**b**) SEM images of CoVO-10-CNT-450P after stability test.

XPS analyses of CoVO-10-CNT and CoVO-10-CNT-450P

Table S1. The element contents of Co, V, O, C and P elements in CoVO-10-CNT CoVO-10-CNT-450P determined by XPS analyses.

Sample	Content	Co	V	O	C	P
CoVO-10-CNT	At%	8.98	6.84	29.35	54.83	0
CoVO-10-CNT-450P	At%	8.38	6.84	27.20	52.49	5.09

Table S2. The resistance values for HER calculated from the EIS results of Figure 5e.

Catalyst	CoVO-10	CoVO-10-CNT	CoVO-10-CNT-400P	CoVO-10-CNT-450P	CoVO-10-CNT-500P
R_s (Ω)	1.38	1.62	1.41	1.32	1.58
R_{ct}(Ω)	8.05	5.27	4.11	2.63	3.12

Table S3. The resistance values for OER calculated from the EIS results of Figure 6e.

Catalyst	CoVO-10	CoVO-10-CNT	CoVO-10-CNT-400P	CoVO-10-CNT-450P	CoVO-10-CNT-500P
R_s (Ω)	2.06	1.62	1.41	1.32	1.58
R_{ct}(Ω)	16.35	11.68	9.69	5.85	7.16

Table S4. Performance comparison of recent non-noble metal bifunctional HER and OER electrocatalysts tested in 1.0 M KOH electrolyte.

**Catalyst**	**η_{HER}/mV vs. RHE 10 mA·cm^{-2}**	**η_{OER}/mV vs. RHE 10 mA·cm^{-2}**	**References**
CoNi/MnO@CNTs	166	275	[13]
Co_3O_4@NiFe-LDH	79	215	[6]
$Co_{5.47}N$ NP@N-PC	149	284	[2]
CoFe LDH-F/NF	166	260	[4]
Zn–Co–S nanoneedle/CFP	234	320	[11]
p-$CoSe_2$/CC	138	243	[9]
CoMo-LDH	115	290	[1]
CoP NS/CC	90	310	[14]
CoP_3 NAs/CFP	119	334	[10]
$Cu_{0.3}Co_{2.7}P$/NC	220	190	[8]
CoP@SNC	174	350	[7]
CoP film	94	345	[3]
$Ni_xCo_{3-x}S_4/Ni_3S_2$/NF	136	160	[12]
CoS_2 HNSs	193	290	[5]
**CoP/V_3P_4-CNT**	**124**	**280**	**This work**

Table S5. Comparison the overall water splitting performance of CoVO-10-CNT-450P with previous reported superior bifunctional electrocatalysts tested in 1.0 M KOH.

Catalytic material	Voltage@10 mA cm-2	Ref.
NiFeS@MXene/NF	1.57	[15]
Ni-Co-S film/Cu foam	1.67	[16]
NiFeRu-LDH	1.52	[17]
NiFe/$NiCo_2O_4$/Ni foam	1.67	[18]
Cu_3P NS/Ni foam	1.67	[19]
Mo_2C@C nanosheets	1.73	[20]
FeCo/Carbon paper	1.68	[21]
**CoVO-10-CNT-450P**	**1.66**	**This work**

References:

1. Bao, J.; Wang, Z.; Xie, J.; Xu, L.; Lei, F.; Guan, M.; Huang, Y.; Zhao, Y.; Xia, J.; Li, H. The CoMo-LDH Ultrathin Nanosheet as a Highly Active and Bifunctional Electrocatalyst for Overall Water Splitting. *Inorg. Chem. Front.* 2018, 5, 2964–2970.
2. Chen, Z.; Ha, Y.; Liu, Y.; Wang, H.; Yang, H.; Xu, H.; Li, Y.; Wu, R. In Situ Formation of Cobalt Nitrides/Graphitic Carbon Composites as Efficient Bifunctional Electrocatalysts for Overall Water Splitting. *ACS. Appl. Mater. Interf.* 2018, 10, 7134–7144.
3. Jiang, N.; You, B.; Sheng, M.; Sun, Y. Electrodeposited Cobalt-Phosphorous-Derived Films as Competent Bifunctional Catalysts for Overall Water Splitting. *Angew. Chem. Int. Ed.* 2015, 54, 6251–6254.
4. Liu, P.F.; Yang, S.; Zhang, B.; Yang, H.G. Defect-Rich Ultrathin Cobalt-Iron Layered Double Hydroxide for Electrochemical Overall Water Splitting. *ACS. Appl. Mater. Interf.* 2016, 8, 34474–34481.
5. Ma, X.; Zhang, W.; Deng, Y.; Zhong, C.; Hu, W.; Han, X. Phase and Composition Controlled Synthesis of Cobalt Sulfide Hollow Nanospheres for Electrocatalytic

Water Splitting. *Nanoscale.* 2018, 10, 4816–4824.

6. Meng, L.; Xuan, H.; wang, J.; Liang, X.; Li, Y.; Yang, J.; Han, P. Flower-Like Co_3O_4@NiFe-LDH Nanosheets Enable High-Performance Bifunctionality Towards Both Electrocatalytic HER and OER in Alkaline Solution. *J. Alloy. Compd.* 2022, 919, 165877.
7. Meng, T.; Hao, Y.-N.; Zheng, L.; Cao, M. Organophosphoric Acid-Derived CoP Quantum Dots@S,N-Codoped Graphite Carbon as a Trifunctional Electrocatalyst for Overall Water Splitting and Zn-Air Batteries. *Nanoscale* 2018, 10, 14613–14626.
8. Song, J.; Zhu, C.; Xu, B.Z.; Fu, S.; Engelhard, M.H.; Ye, R.; Du, D.; Beckman, S.P.; Lin, Y. Bimetallic Cobalt-Based Phosphide Zeolitic Imidazolate Framework: CoPx Phase-Dependent Electrical Conductivity and Hydrogen Atom Adsorption Energy for Efficient Overall Water Splitting. *Adv. Energy. Mater.* 2017, 7, 1601555.
9. Wan, S.; Jin, W.; Guo, X.; Mao, J.; Zheng, L.; Zhao, J.; Zhang, J.; Liu, H.; Tang, C. Self-Templating Construction of Porous $CoSe_2$ Nanosheet Arrays as Efficient Bifunctional Electrocatalysts for Overall Water Splitting. *ACS. Sustain. Chem. Eng.* 2018, 6, 15374–15382.
10. Wu, T.; Pi, M.; Zhang, D.; Chen, S. 3D Structured Porous CoP_3 Nanoneedle Arrays as an Efficient Bifunctional Electrocatalyst for the Evolution Reaction of Hydrogen and Oxygen. *J. Mater. Chem. A* 2016, 4, 14539–14544.
11. Wu, X.; Han, X.; Ma, X.; Zhang, W.; Deng, Y.; Zhong, C.; Hu, W. Morphology-Controllable Synthesis of Zn-Co-Mixed Sulfide Nanostructures on Carbon Fiber Paper Toward Efficient Rechargeable Zinc-Air Batteries and Water Electrolysis. *ACS. Appl. Mater. Inter.* 2017, 9, 12574–12583.
12. Wu, Y.; Liu, Y.; Li, G.-D.; Zou, X.; Lian, X.; Wang, D.; Sun, L.; Asefa, T.; Zou, X. Efficient Electrocatalysis of Overall Water Splitting by Ultrasmall $Ni_xCo_{3-x}S_4$ Coupled Ni_3S_2 Nanosheet Arrays, *Nano. Energy.* 2017, 35, 161–170.
13. Zhou, C.; Han, X.; Zhu, F.; Zhang, X.; Lu, Y.; Lang, J.; Cao, X.; Gu, H. Facile Synthesis of the Encapsulation of Co-Based Multimetallic Alloys/Oxide Nanoparticles Nirtogen-Doped Carbon Nanotubes as Electrocatalysts for the

HER/OER. *Int. J. Hydrogen. Energ.* 2022, 47, 27775–27786.

14. Zhu, W.; Zhang, W.; Li, Y.; Yue, Z.; Ren, M.; Zhang, Y.; Saleh, N.M.; Wang, J. Energy-Efficient 1.67 V Single and 0.90 V Dual-Electrolyte Based Overall Water-Electrolysis Devices Enabled by a ZIF-L Derived Acid-Base Bifunctional Cobalt Phosphide Nanoarray. *J. Mater. Chem. A.* 2018, 6, 24277–24284.
15. Chanda, D.; Kannan, K.; Gautam, J.; Meshesha, M. M.; Jang, S. G.; Dinh, V. A.; Yang, B. L., Effect of the Interfacial Electronic Coupling of Nickel-Iron Sulfide Nanosheets with Layer Ti_3C_2 MXenes as Efficient Bifunctional Electrocatalysts for Anion-Exchange Membrane Water Electrolysis. *Applied Catalysis B: Environmental* 2023, 321, 122039.
16. Liu, T.; Sun, X.; Asiri, A. M.; He, Y., One-Step Electrodeposition of Ni–Co–S Nanosheets Film as a Bifunctional Electrocatalyst for Efficient Water Splitting. *Int. J. Hydrogen Energy* 2016, 41, 7264–7269.
17. Chen, G.; Wang, T.; Zhang, J.; Liu, P.; Sun, H.; Zhuang, X.; Chen, M.; Feng, X., Accelerated Hydrogen Evolution Kinetics on NiFe-Layered Double Hydroxide Electrocatalysts by Tailoring Water Dissociation Active Sites. *Adv. Mater*. 2018, 30, 1706279.
18. Xiao, C.; Li, Y.; Lu, X.; Zhao, C., Bifunctional Porous NiFe/$NiCo_2O_4$/Ni Foam Electrodes with Triple Hierarchy and Double Synergies for Efficient Whole Cell Water Splitting. *Adv. Funct. Mater*. 2016, 26, 3515–3523.
19. Han, A.; Zhang, H.; Yuan, R.; Ji, H.; Du, P., Crystalline Copper Phosphide Nanosheets as an Efficient Janus Catalyst for Overall Water Splitting. *ACS Appl. Mater. Interfaces* 2017, 9, 2240–2248.
20. Wang, H.; Cao, Y.; Sun, C.; Zou, G.; Huang, J.; Kuai, X.; Zhao, J.; Gao, L., Strongly Coupled Molybdenum Carbide on Carbon Sheets as a Bifunctional Electrocatalyst for Overall Water Splitting. *ChemSusChem* 2017, 10, 3523–3523.
21. Liu, W.; Du, K.; Liu, L.; Zhang, J.; Zhu, Z.; Shao, Y.; Li, M., One-Step Electroreductively Deposited Iron-Cobalt Composite Films as Efficient Bifunctional Electrocatalysts for Overall Water Splitting. *Nano Energy* 2017, 38, 576–584.
